# Total Flavonoids of *Apocynum venetum* Ameliorate High-Fat Diet-Induced Lipid Accumulation in Mice and Hepatocytes by Activating the AMPK Signaling Pathway

**DOI:** 10.3390/nu18101586

**Published:** 2026-05-16

**Authors:** Wennu Tang, Wenchang Ding, Lu Deng, Dong Wang, Haixia Wang, Yu Li, Rulin Ma

**Affiliations:** 1 School of Public Health, Shihezi University, Shihezi 832000, China; wilon09@163.com (W.T.); a1658366476@163.com (W.D.);; 2 Key Laboratory for Prevention and Control of Emerging Infectious Diseases and Public Health Security, The Xinjiang Production and Construction Corps, Shihezi 832000, China; 3 Key Laboratory of Xinjiang Endemic and Ethnic Diseases, Ministry of Education, School of Medicine, Shihezi University, Shihezi 832000, China

**Keywords:** metabolic dysfunction-associated steatotic liver disease, total flavonoids of *Apocynum venetum*, AMPK signaling pathway, lipid metabolism, hepatic steatosis, high-fat diet

## Abstract

**Objectives**: Metabolic dysfunction-associated steatotic liver disease (MASLD) is characterized by abnormal hepatic lipid accumulation and is frequently driven by factors such as a high-fat diet (HFD). Total flavonoids of *Apocynum venetum* (TFAV), the bioactive constituents of a traditional medicinal plant, have demonstrated antioxidant and lipid-modulating properties. However, their therapeutic potential against MASLD and the underlying mechanisms are not explored. This study aims to evaluate the ameliorative effects of TFAV on HFD-induced MASLD utilizing both in vivo animal and in vitro cellular models. **Methods**: C57BL/6J were allocated to control, high-fat diet (HFD), TFAV (100 mg/kg/day), and TFAV intervention groups (25, 50, and 100 mg/kg/day). In vitro, WRL68 hepatocytes were stimulated with free fatty acids (FFAs) to establish a cellular model of steatosis. Liver function, serum lipid profiles, hepatic histopathology, and the AMPK signaling pathway were assessed. **Results**: TFAV intervention significantly improved serum biochemical profiles in the animal models; for instance, co-treatment with 100 mg/kg/day TFAV and HFD reduced TC, TG, and LDL-C levels by 20.59%, 45.26%, and 38.24% respectively (*p* < 0.05), and effectively alleviated hepatic steatosis and hepatocyte ballooning. Furthermore, TFAV markedly inhibited intracellular reactive oxygen species (ROS) levels and activated the AMPK signaling pathway (*p* < 0.05). This was accompanied by the downregulation of SREBP-1c and ACC expression (*p* < 0.05), as well as the upregulation of ATGL and CPT1α expression (*p* < 0.05). **Conclusions**: These results demonstrates that TFAV remodel hepatic lipid homeostasis by activating the AMPK signaling pathway, and exerting significant preventive and protective effects against the progression of HFD-induced MASLD in vivo.

## 1. Introduction

Metabolic dysfunction-associated steatotic liver disease (MASLD) is the most prevalent chronic liver disorder globally, characterized by excessive hepatic lipid accumulation [1]. The disease spectrum ranges from simple steatosis to metabolic dysfunction-associated steatohepatitis (MASH), which can progress to cirrhosis and hepatocellular carcinoma (HCC) [2]. Currently, the global prevalence of MASLD has exceeded 30% of the population [3].

The core pathophysiological hallmark of MASLD is the aberrant accumulation of lipids within hepatocytes [4]. Adenosine monophosphate-activated protein kinase (AMPK) is a pivotal cellular energy sensor that can mitigate lipid dysregulation by modulating lipid metabolism [5]. AMPK activation effectively suppresses the lipogenic pathway by directly phosphorylating SREBP-1c, locking it in an inactive precursor state and thereby inhibiting its transcriptional activity [6]. Adipose triglyceride lipase (ATGL) is the rate-limiting enzyme for triglyceride hydrolysis in lipid droplets, and its expression is inversely correlated with hepatic fat content [7]. Carnitine palmitoyltransferase 1α (CPT1α) is the rate-limiting enzyme for mitochondrial fatty acid oxidation (FAO) [8]. The activation of AMPK upregulates the expression and activity of both ATGL, and CPT1α [9,10], thereby accelerating lipolysis and fatty acid oxidation and facilitating fatty acid clearance. AMPK not only suppresses lipogenesis but also promotes lipolysis and energy expenditure, thereby synergistically restoring lipid homeostasis. Consequently, the activation of AMPK has emerged as a highly attractive upstream therapeutic strategy for the management of MASLD [11]. Compound C (dorsomorphin) is a well-established, highly selective, and cell-permeable inhibitor of AMPK. It is frequently employed to validate the central role of the AMPK pathway in mediating the metabolic regulatory effects of pharmacological agents [12,13,14].

*Apocynum venetum* L. is a traditional medicinal plant whose primary bioactive constituents are recognized as total flavonoids of *Apocynum venetum* (TFAV) [15]. In addition to its traditional medicinal use, Apocynum venetum is increasingly valued as a functional food and dietary nutrient source. The plant contains a variety of essential trace minerals, including potassium, calcium, iron, and zinc, as well as beneficial amino acids and polysaccharides. Its health-promoting properties are primarily attributed to an exceptionally rich profile of secondary metabolites, comprising over 170 identified compounds, among which flavonoids (e.g., quercetin, isoquercitrin, rutin, and hyperoside) and phenolic acids (e.g., chlorogenic and neochlorogenic acids) are the most prominent. These bioactive constituents exhibit high bioavailability and exert potent antioxidant effects by capturing free radicals and upregulating endogenous antioxidant enzymes [16]. Thus, regular consumption of *A. venetum* as a functional beverage provides meaningful health benefits, particularly in cardiovascular protection, lipid lowering, hepatoprotection, and the mitigation of oxidative stress-related disorders. A study reported that, *Apocynum venetum* demonstrated significant preliminary potential in lowering blood lipids and ameliorating hepatic steatosis [17]. Furthermore, research studies have reported that TFAV derived from leaves markedly alleviated hepatic steatosis and oxidative stress levels in rats with high-fat diet (HFD)-induced hyperlipidemia [18]. Similarly, In a study investigating the flavonoids derived from *Apocynum venetum* leaves, a reduction in lipid metabolism-related factors was also detected in rat serum [19]. Flavonoids are a class of natural polyphenols widely distributed in plants. Several flavonoid monomers, such as quercetin, have been validated as potent AMPK activators [20,21,22] and have shown beneficial effects in various metabolic disease models [23,24]. A study [25] demonstrated that the aqueous extract of *Apocynum venetum* L. and isoquercitrin significantly attenuated body weight gain and reduced plasma triglyceride (TG) and total cholesterol (TC) levels in HFD-induced obese mice; importantly, these anti-obesity effects were blunted by inhibitors of AMPK and SREBP-1c. Therefore, this research was designed to investigate the effects of TFAV in C57BL/6J mice exposed to HFD-induced metabolic stress and in a free fatty acid (FFA)-induced hepatocyte steatosis model, Our objective was to evaluate the efficacy of TFAV in ameliorating MASLD and to elucidate the pivotal role of the AMPK-related signaling pathway as a key underlying mechanism.

## 2. Materials and Methods

### 2.1. TFAV

TFAV utilized in this study were obtained as a standardized extract from Xi’an Ebos Biotech Co., Ltd. (Xi’an, China; Batch No. Ebos-231025). According to the manufacturer’s quantitative assay, the total bioflavonoid content of the extract was 90.25%. As a highly enriched botanical fraction rather than an absolute pure compound, the extract effectively concentrates flavonoids while eliminating the vast majority of non-flavonoid constituents (such as high-polarity phenolic acids and polysaccharides) [26].

### 2.2. Animal Experiments

To investigate the in vivo protective effects of TFAV against MASLD and to evaluate its dose-response efficacy, we established a diet-induced obesity murine model. 48 specific pathogen-free (SPF) male C57BL/6J mice (6 weeks old, weighing 18 ± 2 g) were obtained from Sibeifu Biotechnology Co., Ltd. (Beijing, China). All animals were acclimatized for one week prior to the commencement of the experiments. The animal study protocol was approved by the Experimental Animal Ethics Committee of the First Affiliated Hospital of Shihezi University (No. A2023-212-01). All animal experiments were carried out in strict agreement with the ethical guidelines issued by the Ethics Committee of the First Affiliated Hospital of Shihezi University.

The mice were randomly allocated into six groups (*n* = 8): normal control (NC) TFAV-only (100 mg/kg/day), HFD model, and HFD + TFAV intervention (25, 50, and 100 mg/kg/day) groups. The NC group served as the baseline, while the HFD model group was established to mimic the pathological progression of human MASLD. TFAV-only group (100 mg/kg/day) was included to assess the basal safety and physiological impact of the extract on healthy mice. The remaining three HFD groups were concurrently treated with varying doses of TFAV (25, 50, and 100 mg/kg/day) to determine its dose-dependent therapeutic efficacy against lipid accumulation. The NC and TFAV-only groups were maintained on a standard rodent chow diet (Code: 1032, Beijing HFK Bioscience Co., Ltd., Beijing, China). This basic diet is formulated with agricultural grains (e.g., corn, wheat bran, and soybean meal) containing ≥20.0% crude protein and ≥4.0% crude fat. The remaining four groups were fed a HFD to induce MASLD. The HFD was a Diet-Induced Obesity series formula (Catalog No. H10060, Beijing HFK Bioscience Co., Ltd.). Compared to the standard chow, the composition of the HFD was heavily modified by the incorporation of lard (providing abundant saturated fatty acids) and sucrose. Specifically, the HFD provided 60% of total calories from fat, 20% from carbohydrates, and 20% from protein, yielding a total energy density of 5.24 kcal/g. Mice in the TFAV intervention groups received their respective doses via daily oral gavage, while the NC and HFD model groups were administered an equivalent volume of physiological saline. The experimental feeding and intervention duration lasted for 16 weeks. The animal laboratory was maintained at 22–26 °C, with approximately 50% relative humidity and a 12-h light/dark cycle. During the experiment, mice were provided unrestricted access to food and water [27].

### 2.3. Cell Experiments

To further elucidate the underlying molecular mechanisms of TFAV, particularly its direct regulatory effects on hepatocytes and the AMPK signaling pathway, we established an in vitro model of hepatic steatosis. The human normal hepatocyte cell line WRL68 was obtained from Thermo Fisher Scientific (Beijing, China) Co., Ltd. Cells were cultured in Minimum Essential Medium supplemented with 10% fetal bovine serum and 1% penicillin-streptomycin, and maintained in a humidified incubator at 37 °C with 5% CO_2_. Subculturing was performed when the cell density reached 80–90% confluence. To simulate the lipotoxic environment and lipid overload characteristic of MASLD in vitro, hepatocytes were stimulated with FFAs. FFA stock solution was prepared by mixing oleic acid and palmitic acid at a 2:1 molar ratio [28]. Cells were exposed to 1 mmol/L FFA to induce steatosis. To determine the optimal intervention dose, cell viability under various TFAV concentrations was first assessed. Subsequently, targeted mechanistic experiments were conducted by treating the FFA-induced steatotic cells with TFAV in the presence or absence of Compound C, a specific AMPK inhibitor. This experimental design allowed us to definitively verify whether the lipid-lowering and antioxidant effects of TFAV are dependent on AMPK activation.

### 2.4. Western Blot (WB) Analysis

Cells were collected, lysed in RIPA buffer, and centrifuged at 12,000× *g* rpm for 10 min at 4 °C. The supernatant was collected, and protein concentration was determined using the BCA assay. Proteins were separated by SDS-PAGE (80 V for 30 min, then 110 V for 90 min) and transferred onto membranes by semi-dry or wet transfer depending on protein molecular weight. Membranes were blocked with a rapid blocking buffer for 15 min and then incubated with primary antibodies at 4 °C for 12 h. After three washes with TBST, membranes were incubated with secondary antibodies at room temperature for 2 h. Protein bands were visualized using ECL chemiluminescence reagents [29]. Primary antibodies against *p-*AMPK, AMPK, SREBP-1c, ACC, ATGL, CPT1α and GAPDH, as well as goat anti-mouse and goat anti-rabbit secondary antibodies, were purchased from Proteintech Group, Inc. (Wuhan, China).

### 2.5. Oil Red O Staining

Samples were fixed with 4% paraformaldehyde for 20–30 min and rinsed with phosphate-buffered saline (PBS). After a 30 s immersion in 60% isopropanol, the samples were incubated in freshly prepared Oil Red O working solution for 10–20 min. After a second rinse with 60% isopropanol, the nuclei were counterstained with Mayer’s hematoxylin for 1–2 min, followed by five washes with distilled water. The samples were then incubated in Oil Red O buffer for 1 min for “blueing”; the buffer was then discarded and replaced with distilled water to cover the cells. Lipid droplets and cellular morphology were observed and imaged under a light microscope [30].

### 2.6. Intracellular TC and TG Content Measurement

Cell pellets were collected and disrupted via ultrasonication to prepare cellular homogenates. The homogenates were subsequently transferred to a 96-well plate, where TC and TG working reagents and calibrators were added according to the manufacturer’s instructions [31,32]. The absorbance was measured at 500 nm using an automated microplate reader, and the concentrations of TC and TG were calculated based on the measured vaules.

### 2.7. Quantitative Real-Time PCR (qRT-PCR) Analysis

The primer sequences for AMPK, SREBP-1c, ATGL, CPT1α, and β-actin were designed based on reference sequences obtained from the NCBI GenBank database, and verified for specificity using the Nucleotide BLAST tool (https://blast.ncbi.nlm.nih.gov/Blast.cgi, accessed on 15 May 2026). After quantifying the total RNA concentration of the samples, cDNA was synthesized using a reverse transcription kit in accordance with the manufacturer’s protocol. Subsequently, qRT-PCR was performed to amplify the target genes. The cycle threshold (Ct) values were recorded, and the relative mRNA expression levels were calculated using the 2^−ΔΔCT^ method, with β-actin serving as the internal control for normalization [33].

### 2.8. Statistical Analysis

Experimental data are expressed as the mean ± standard deviation (SD). Statistical analyses and data visualization were performed using SPSS software (version 20.0) and GraphPad Prism (version 9.0). Differences among multiple groups were evaluated using one-way analysis of variance (ANOVA), followed by the Student–Newman–Keuls (SNK) test for pairwise comparisons. The threshold for statistical significance was set at 0.05, and *p* < 0.05 was considered to indicate a significant difference.

## 3. Results

### 3.1. TFAV Ameliorates HFD-Induced Hepatic Lipid Accumulation and Metabolic Dysfunction in Mice

**Figure 1:**
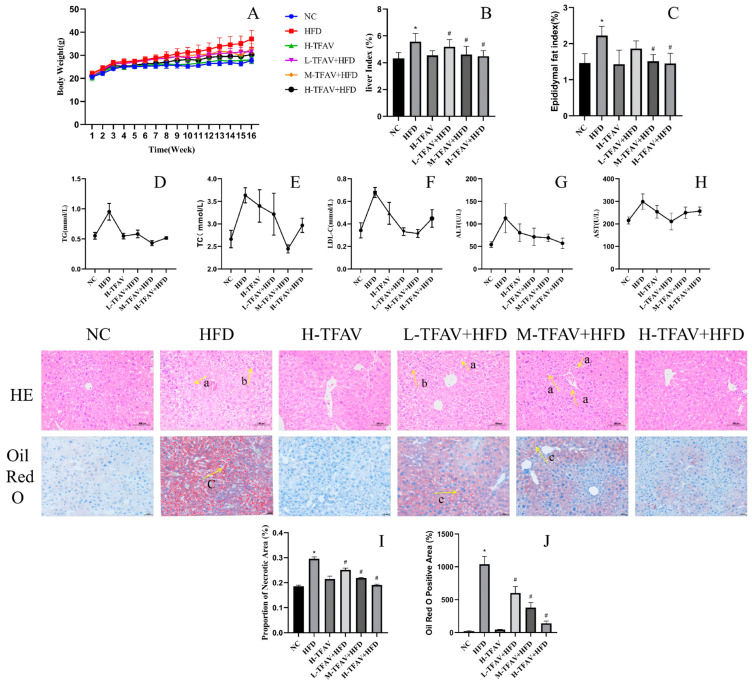
Effects of TFAV on HFD-induced hepatic lipid accumulation in mice. (**A**) Body weight changes; (**B**) Liver index; (**C**) Epididymal fat index; (**D**) Serum TG levels; (**E**) Serum TC levels; (**F**) Hepatic LDL-C levels; (**G**) Serum ALT levels; (**H**) Serum AST levels; (**I**) Representative H&E staining of liver sections, arrow a indicates lipid droplets, and arrow b indicates hepatocyte ballooning; (**J**) Representative Oil Red O staining of liver sections, arrow c indicates lipid droplets; *n* = 8 per group; compared with NC group, * *p* < 0.05; compared with HFD group, ^#^ *p* < 0.05.

Following 16 weeks of high-fat diet (HFD) feeding, mice in the HFD group exhibited rapid weight gain. Body weight in all TFAV-treated groups showed statistically significant disparities compared to both the NC and HFD groups (NC 27.75 ± 1.12, L+H 31.55 ± 1.99, M+H 32.98 ± 2.13, H+H 30.29 ± 3.02 vs. HFD 37.09 ± 3.68, *p* < 0.05) (Figure 1A). Moreover, The Liver Index is used to assess the degree of hepatic hypertrophy or lipid accumulation in mice. It is typically calculated as the ratio of liver weight to total body weight, expressed as a percentage. The liver index and epididymal fat index in the HFD group were significantly reduced in the medium and high-dose TFAV+HFD groups (liver Index: 5.57 ± 0.62 vs. 4.62 ± 0.61, 4.49 ± 0.42, *p* < 0.05; epididymal fat index: 2.22 ± 0.26 vs. 1.51 ± 0.19, 1.45 ± 0.28, *p* < 0.05) (Figure 1B,C).

Serum biochemical analysis (Figure 1D–H) showed that TC, TG, low-density lipoprotein cholesterol (LDL-C), alanine aminotransferase (ALT), and aspartate aminotransferase (AST) levels were markedly elevated in the HFD group relative to the NC (TC: 3.74 ± 0.19 vs. 2.67 ± 0.24, *p* < 0.05; TG: 0.95 ± 0.44 vs. 0.55 ± 0.16, *p* < 0.05; LDL-C: 0.68 ± 0.14 vs. 0.34 ± 0.20, *p* < 0.05; ALT: 138.10 ± 19.68 vs. 54.32 ± 3.17, *p* < 0.05; AST: 291.50 ± 58.76 vs. 205.40 ± 18.31, *p* < 0.05). In contrast, TFAV administration at all three doses significantly reduced the serum levels of TC, TG, LDL-C, ALT, and AST compared with the HFD group (TC: 3.74 ± 0.19 vs. 3.26 ± 0.58, 2.45 ± 0.16, 2.97 ± 0.22, *p* < 0.35; TG: 0.95 ± 0.44 vs. 0.58 ± 0.20, 0.43 ± 0.13, 0.52 ± 0.06, *p* < 0.05; LDL-C: 0.68 ± 0.14 vs. 0.33 ± 0.10, 0.32 ± 0.11, 0.42 ± 0.14, *p* < 0.05; ALT: 138.10 ± 19.68 vs. 60.72 ± 13.22, 57.38 ± 4.20, 55.68 ± 5.17, *p* < 0.05; AST: 291.50 ± 58.76 vs. 197.60 ± 41.68, 259.20 ± 34.39, 272.70 ± 19.25, *p* < 0.05).

Histopathological assessment by H&E (Figure 1I) and Oil Red O (Figure 1J) staining revealed markedly greater lipid droplet accumulation in the livers of HFD-fed mice than in NC group, accompanied by evident hepatocyte ballooning (*p* < 0.05). These findings indicate successful establishment of MASLD model characterized with severe hepatic steatosis and demonstrate that TFAV treatment effectively ameliorated these pathological manifestations.

### 3.2. TFAV Reduces FFA-Induced Lipid Accumulation and Oxidative Stress in Hepatocytes

**Figure 2:**
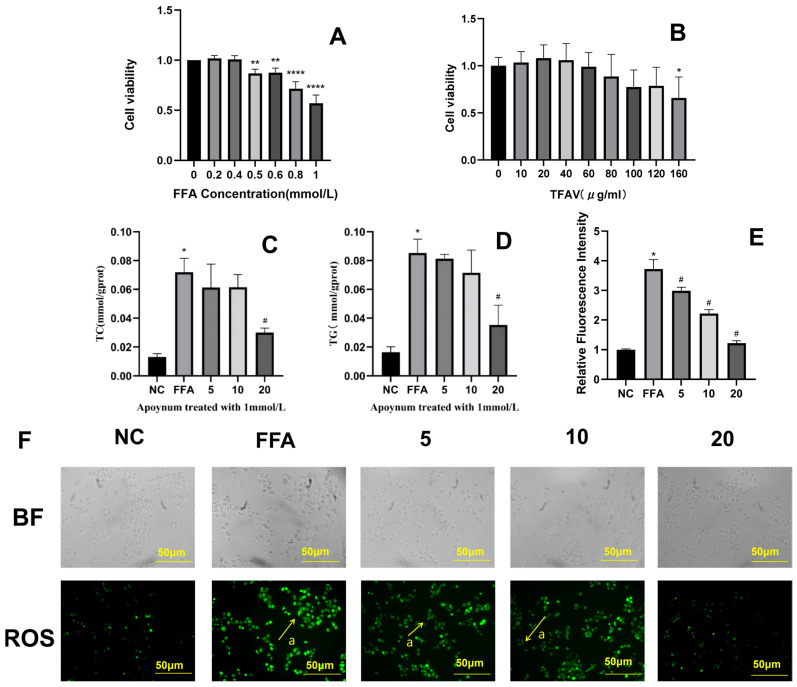
TFAV reduces FFA-induced lipid accumulation and oxidative stress in hepatocytes. (**A**) Effects of various FFA concentrations on cell viability; (**B**) Effects of various TFAV concentrations on cell viability; (**C**) Intracellular TC levels; (**D**) Intracellular TG levels; (**E**,**F**) Effects of TFAV on ROS levels in FFA-induced WRL68 cells, arrow a indicates DCF(ROS),BF stands for bright field. *n* = 3 per group; * *p* < 0.05, ** *p* < 0.01, **** *p* < 0.0001; compared with NC group, * *p* < 0.05; compared with FFA group, ^#^ *p* < 0.05.

To establish an in vitro hepatic steatosis model, WRL68 cells were treated with FFAs. Compared with the NC group, cell viability decreased significantly when the FFA concentration exceeded 0.5 mmol/L (*p* < 0.05), reaching approximately 50% at a concentration of 1 mmol/L (Figure 2A). Therefore, 1 mmol/L FFA was selected to induce steatosis [34]. Assessment of TFAV at eight concentrations (10–160 µg/mL) showed that cell viability began to decline when concentrations exceeded 20 µg/mL compared to the control (Figure 2B). Thus, 20 µg/mL TFAV was selected as the optimal intervention dose.

Compared with the NC group, FFA treatment significantly elevated intracellular TC (Figure 2C) and TG levels (Figure 2D) (TC: 0.07 ± 0.01 vs. 0.01 ± 0.002, *p* < 0.05; TG: 0.085 ± 0.01 vs. 0.02 ± 0.004, *p* < 0.05), confirming the successful establishment of the lipid-overload cell model. Notably, the administration of 20 µg/mL TFAV significantly reversed the increases in TC and TG (TC: 0.07 ± 0.01 vs. 0.03 ± 0.003, *p* < 0.05; TG: 0.085 ± 0.01 vs. 0.35 ± 0.014, *p* < 0.05). Moreover, the FFA group exhibited markedly increase in intracellular reactive oxygen species (ROS) fluorescence intensity compared to the NC group (*p* < 0.05) (Figure 2E). Whereas TFAV treatment significantly suppressed ROS levels compared with the FFA group (*p* < 0.05).

### 3.3. Effects of TFAV on the AMPK Signaling Pathway and Lipid Metabolism-Related Protein Expression

**Figure 3:**
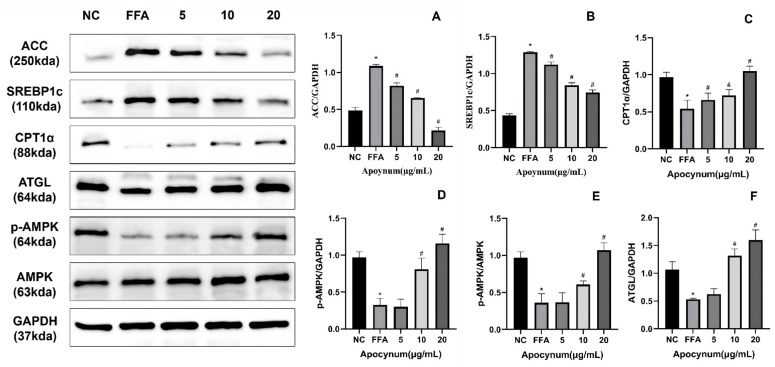
Effects of TFAV on the AMPK Signaling Pathway and Lipid Metabolism-Related Protein Expression. (**A**) ACC relative protein expression; (**B**) SREBP-1c relative protein expression; (**C**) CPT1α relative protein expression; (**D**,**E**) Activation of the AMPK signaling pathway and the quantified *p-*AMPK/AMPK ratio; (**F**) ATGL relative protein expression. *n* = 3 per group; compared with NC group, * *p* < 0.05; compared with FFA group, ^#^ *p* < 0.05.

Compared with the NC group, the FFA group exhibited a significant decrease in the *p-*AMPK/AMPK ratio (Figure 3D,E) (*p* < 0.05), and downregulated expression of ATGL (Figure 3F) and CPT1α (Figure 3C) (*p* < 0.05). In contrast, the expression levels of lipogenic proteins, including SREBP-1c (Figure 3B) and ACC (Figure 3A), were significantly upregulated in the FFA group (*p* < 0.05). Compared with the FFA group, treatment with 10 and 20 μg/mL TFAV significantly increased the *p-*AMPK/AMPK ratio and upregulated ATGL and CPT1α expression (*p* < 0.05). Moreover, TFAV at all tested concentrations significantly reduced the protein expression of SREBP-1c and ACC (*p* < 0.05).

### 3.4. Compound C Blunts the Regulatory Effects of TFAV on the AMPK Pathway

**Figure 4:**
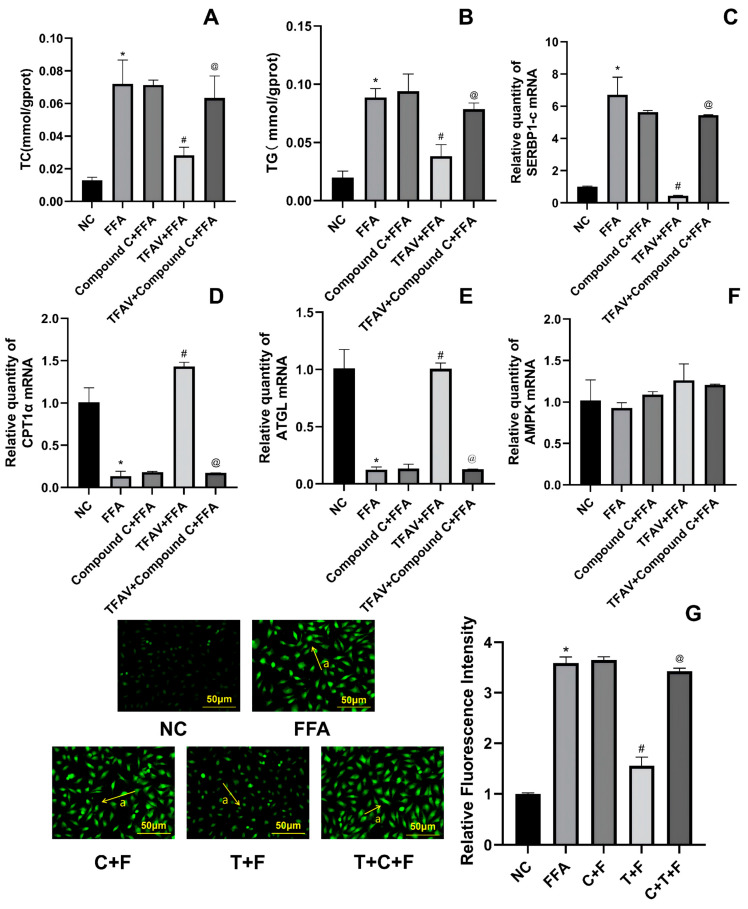
Compound C Blunts the Regulatory Effects of TFAV on the AMPK Pathway. (**A**,**B**) Intracellular TC and TG levels; (**C**) SREBP-1c relative mRNA expression; (**D**) ATGL relative mRNA expression; (**E**) CPT1α relative mRNA expression; (**F**) AMPK relative mRNA expression; (**G**) Cellular ROS levels, arrow a indicates DCF(ROS). *n* = 3 per group; compared with NC group, * *p* < 0.05; compared with FFA group, ^#^ *p* < 0.05; compared with TFAV+FFA group, ^@^ *p* < 0.05.

To further validate the necessity of AMPK activation in TFAV-mediated protection, we employed the specific inhibitor Compound C. Compared with the TFAV + FFA group, the TFAV + Compound C + FFA group exhibited a significant recovery in intracellular TC (Figure 4A) and TG (Figure 4B) levels (TC: 0.03 ± 0.005 vs. 0.06 ± 0.013, *p* < 0.05; TG: 0.04 ± 0.01 vs. 0.08 ± 0.005, *p* < 0.05), as well as SREBP-1c mRNA expression (*p* < 0.05) (Figure 4C). This trend was consistent with the observed changes in ROS levels (*p* < 0.05) (Figure 4G). In contrast, the mRNA expression levels of ATGL (Figure 4D) and CPT1α (Figure 4E) were significantly downregulated following Compound C treatment (*p* < 0.05). Notably, there were no statistically significant differences in TC and TG levels between the TFAV + Compound C + FFA group and the FFA group (TC: 0.06 ± 0.013 vs. 0.07 ± 0.015, *p* > 0.05; TG: 0.08 ± 0.005 vs. 0.09 ± 0.008, *p* > 0.05). Furthermore, no significant alterations in AMPK mRNA levels (Figure 4F) were observed across all groups (*p* > 0.05).

## 4. Discussion

MASLD has emerged as the leading chronic liver disease worldwide, driving the development of cirrhosis, HCC, and associated metabolic complications [35]. Its core pathological basis is rooted in profound dysregulation of hepatic lipid homeostasis, wherein the uptake and de novo synthesis of FFAs markedly outpace the hepatic capacity for FAO and lipid export, thereby triggering lipotoxicity and oxidative stress [36]. Discovering natural bioactive compounds that restore lipid homeostasis through multi-targeted mechanisms remains a key priority in current pharmacological research [37]. Chen et al. performed detailed quantification using ultra-fast liquid chromatography tandem triple quadrupole mass spectrometry (UFLC-MS/MS), and identified hyperoside/isoquercitrin and quercetin-3-O-sophoroside as the predominant bioactive compounds in *Apocynum venetum* L. extracts [38]. A study has confirmed that flavonoids from *Apocynum venetum* can drive the activation of the AMPK pathway [39]. Our study provides systematic evidence that TFAV markedly ameliorate MASLD by specifically activating the AMPK signaling pathway, thereby rebalancing lipid anabolism and catabolism and restoring hepatic lipid homeostasis.

A study reported that the inhibition of AMPK signaling abolished the protective effects of salidroside against lipid accumulation in both hepatocytes and liver tissue induced by high-fat and high-cholesterol stimuli [40]. Similarly, the present study demonstrated that TFAV intervention significantly suppressed excessive weight gain in HFD-fed mice and improved serum lipid profiles, as evidenced by reduced levels of TC, TG, and LDL-C. These findings align with recent research highlighting the efficacy of plant-derived flavonoids in mitigating metabolic syndrome [41,42]. Histopathologically, TFAV markedly attenuated hepatocellular steatosis and ballooning degeneration, accompanied by a reduction in serum ALT and AST levels; these findings are consistent with the results reported by Zhang et al. [19]. Our quantitative analysis demonstrated that TFAV (100 mg/kg) reduced hepatic TC and TG levels by 20.59% and 45.26%, respectively, which was closely associated with a increase in AMPK phosphorylation. These results provide robust evidence for the lipid-lowering efficacy of TFAV.

Our results demonstrated that FFA suppressed hepatic AMPK activity by maintaining AMPK in a dephosphorylated state, whereas TFAV intervention significantly increased the *p-*AMPK/AMPK ratio in both liver tissues and hepatocytes, indicating activation of the AMPK signaling pathway. No significant differences in AMPK mRNA levels were observed among all experimental groups following Compound *C*-mediated AMPK inhibition; however, the *p-*AMPK/AMPK ratio and *p-*AMPK protein expression were markedly elevated by TFAV treatment. This observation suggests that TFAV-mediated AMPK activation occurs at the post-translational level, specifically through phosphorylation modification, rather than at the transcriptional level. This regulatory mode is widely prevalent among flavonoids. For instance, A study reported in 2025 that flavonoids and other natural compounds activate AMPK primarily by enhancing phosphorylation at the Thr172 residue, rather than by altering the transcriptional expression of PRKAA1/2 (the genes encoding AMPK) [11]. Similarly, Wang et al. found that flavonoids can maintain the phosphorylated state of AMPK by inhibiting the activity of phosphatases (e.g., PP2A) [43], or by promoting AMPK phosphorylation via its upstream kinase LKB1 [44].

Shi et al. reported that pharmacological AMPK inhibition suppressed Ad-NLRP6-induced lipolysis and FAO in hepatocytes, thereby aggravating lipid deposition [45]. Similarly, Xu et al. employed Compound C to inhibit AMPK and demonstrated that AMPK alleviates hepatic steatosis by modulating the SREBP-1c signaling pathway [46]. These findings are consistent with our results, which showed that activated AMPK inhibited SREBP-1c and its downstream target ACC through phosphorylation, thereby suppressing de novo lipogenesis. Moreover, TFAV upregulated ATGL and CPT1α expression, promoting lipolysis and mitochondrial fatty acid β-oxidation. Collectively, these data indicate that TFAV restores hepatic lipid homeostasis through a dual regulatory mechanism that concurrently inhibits lipid synthesis and promotes lipolysis and fatty acid oxidation.

Mitochondrial dysfunction and ROS burst triggered by excess hepatic lipid accumulation, which are are pivotal drivers of the progression from MASLD to MASH [47]. Palomer et al. [48] demonstrated that AMPK restores lipid homeostasis by phosphorylating ACC1 and inhibiting SREBP-1c, which enhances mitochondrial β-oxidation efficiency and significantly attenuates endoplasmic reticulum (ER) stress and apoptosis induced by lipid peroxidation. These findings align with our results, which confirmed that TFAV treatment markedly reduced intracellular ROS levels. Crucially, this antioxidant effect was abrogated by AMPK inhibition with Compound C. This indicates that the antioxidant effect of TFAV depends primarily on AMPK-mediated restoration of lipid homeostasis, rather than on direct free radical scavenging, which is consistent with previous findings reported by Chang et al. [49]. Moreover, Suttawong et al. reported that natural bioactive extracts ameliorate fibrosis in high-sugar, HFD-induced MASH by targeting inflammatory and fibrogenic pathways [50]. By reducing hepatic lipid burden, TFAV holds the potential to block the activation of the downstream inflammatory signaling axis, such as the NF-κB pathway, thereby preventing the progression of hepatic fibrosis.

There are several limitations in our study. TFAV is a complex botanical extract, our study did not pinpoint the precise active monomer responsible for the observed therapeutic effects. The specific bioactive constituents playing a core role (e.g., quercetin or isoquercitrin) warrant further isolation and validation. Furthermore, the mechanistic validation of the AMPK pathway in this study relied solely on the pharmacological inhibitor Compound C in vitro. Future investigations utilizing liver-specific AMPK knockout murine models are essential to definitively substantiate the in vivo targeted mechanisms of TFAV.

## 5. Conclusions

In summary, our study demonstrates that TFAV significantly ameliorates MASLD and identified the AMPK pathway as the key molecular mechanism. TFAV restored hepatic lipid homeostasis by activating the AMPK signaling pathway to suppress de novo lipogenesis and promote lipid catabolism and FAO, thereby effectively alleviating lipotoxicity-driven oxidative stress. These findings provide a theoretical foundation for the development of TFAV as a natural bioactive agent for the prevention of MASLD and highlight its considerable potential for large-scale industrial application. TFAV could be developed into standardized functional food ingredients or adjunct therapeutic supplements aimed at preventing or attenuating MASLD progression in high-risk populations. Future clinical trials are warranted to validate its long-term safety and efficacy in humans, thereby facilitating its broader use in public health nutrition.

## Data Availability

The raw data supporting the conclusions of this article will be made available by the authors without undue reservation.

## Abbreviations

The following abbreviations are used in this manuscript:

MASLD	Metabolic dysfunction-associated steatotic liver disease
MASH	Directory of open access journals
HCC	Hepatocellular carcinoma
TC	Total cholesterol
TG	Triglyceride
ALT	Alanine aminotransferase
AMPK	Adenosine monophosphate-activated protein kinase
AST	Aspartate aminotransferase
ATGL	Adipose triglyceride lipase
BCA	Bicinchoninic acid
CPT1α	Carnitine palmitoyltransferase 1α
FAO	Fatty acid oxidation
TFAV	Total flavonoids of Apocynum venetum
HFD	High-fat diet
FFA	Free fatty acid
NC	Normal control
PBS	Phosphate-buffered saline
LDL-C	Low-density lipoprotein cholesterol
RIPA	Radio immunoprecipitation assay lysis buffer
ROS	Reactive oxygen species
SDS-PAGE	Sodium dodecyl sulfate polyacrylamide gel electrophoresis
